# Progress in Bacteriology

**Published:** 1903-11-28

**Authors:** 


### PROGRESS IN BACTERIOLOGY.

**Human and Bovine Tuberculosis.**—A summary of Professor Behring's views on the origin and prevention of consumption is given in the *British Medical Journal.*[1] He states his belief that latent tubercle is extremely common in human beings, and may be lighted up when circumstances and surroundings are suitable; thus arise many cases which are erroneously attributed to personal infection by inha ation. Behring attaches little importance to heredity, but believes the mainspring of the disease to be the milk diet of infancy, for at this age the alimentary tract is incapable of resisting the passage of many foreign and noxious substances. The two factors which go to increase the vulnerability are the incomplete epithelial lining of the intestinal tract in infancy, and the absence of active ferment secretion. With regard to the former, experiment has shown that the large albuminous molecules of tetanus and diphtheria antitoxin pass through the intestinal epithelium without being converted into peptones, and anthrax and tubercle bacilli are similarly equally permeable. Guinea-pigs when newly born, if fed with tubercle bacilli, speedily become tuberculous, but later on in life it is extremely difficult to infect them in this way. Bacilli persist longer in the cæcum than in the rest of the alimentary tract. All milk bacteria very readily get into the general circulation. Other pathological causes, such as the exanthemata (particularly measles), abrasion, catarrh, and injury to the intestinal tract produce increased permeability. The tubercle bacillus may, in Behring's opinion, remain latent in the human body for years, or even decades. It is most frequently introduced through the alimentary system. Bovine turberculosis is, too, a very widely spread disease, and resembles human tuberculosis in the fact that it may lie dormant for years. Cattle may be immunised by inoculation with attenuated cultures of living human tubercle bacilli, and this is a strong argument in favour of the identity of the two organisms. All types of tubercle bacilli are not alike, and all human tubercle bacilli are not capable of producing Perlsucht. Behring, however, strongly maintains that tuberculosis in the human and in cattle is one and the same disease, and that the commonest route of infection is through the alimentary canal in early infancy. Max Wolff[2] has induced Perlsucht in a calf by inoculation with material obtained from a primary case of abdominal tuberculosis. The result when human tubercular sputum was employed was doubtful, and Möller's attempts to excite general tuberculosis in cows by the inhalat[illegible] injection, or inoculation of human tuberculous matt[illegible] were all failures. Raw[3] agrees with Koch that bovine tuberculosis is a distinct disease from human tuberculosis, but is of opinion that man is attacked by two distinct species of tubercle, one conveyed by infection from one person to another, and the other by receiving into the body bovine bacilli in milk and meat. He points out how rarely strumous or tuberculous joints, enlarged glands, spinal disease and abdominal tuberculosis are seen arising in adults, and how seldom they are associated with phthisis pulmonalis. The former affections he regards as strictly due to the bovine bacillus introduced during the milk-feeding stage of infancy. Whilst the death-rate from pulmonary tuberculosis has steadily declined that from abdominal tuberculosis has remained stationary or increased, and this is accounted for by the fact that our dairy herds have an increasingly high percentage of tuberculous cows. Whilst the human cannot be distinguished from the bovine tubercle bacillus by microscopical inspection alone, they have persistent and constant peculiarities of growth and morphology. Bacilli obtained by Raw from cases of strumous knee-joint disease, and tabes mesenterica resembled the Perlsucht bacillus, and when inoculated into calves set up tuberculosis of the mesenteric glands. Macfadyen and McConkey's experiments showed that tubercle bacilli are present in 25 per cent. of all children, if cultures be taken from the mesenteric glands. Enlarged cervical glands are due to the invasion of the tissues by bovine tubercle bacilli taken in with food, and if the material from such glands be inoculated into calves, they produce a general tubercular infection.

**Cystitis, Pyelitis, and Pyelonephritis.**—Brown[4] gives the results of his bacteriological examinations in a hundred cases. The urine was collected from the bladder and ureters with strict antiseptic precautions. In pyelitis the amount of albumen in the urine is high and in cystitis it is low. The organism most commonly found is the B. coli, which may be in pure culture, and of variable virulence. Next, in order of frequency, come the tubercle bacillus, staphylococci, B. proteus, B. pyocyaneus, and B. typhosus. Infection from B. coli is commonest in women, the predisposing causes being anæmia, malnutrition, pressure on and injury to the bladder, and any cause obstructing the outflow of urine. The bladder is usually infected through the urethra. Of the cases examined, about one half of the kidney infections had spread up from the bladder, and the remaining half from the blood and lymph streams. Acid urine is more common than alkaline both in cystitis and pyelitis. Tubercle of the kidney is often found independent of tubercle elsewhere.

**Bacteriology of the Eye.**—Randolph[5] gives the following list of micro-organisms as found capable of producing eye inflammations: streptococcus, staphylcoccus, pneumococcus, gonococcus, diplobacillus of Morax-Axenfeld, B. diphtheriæ, B. xerosis, B. tuberculosis, B. pyocyaneous, and B. coli. The lesions excited by the staphylococcus, B. coli, and B. xerosis are usually mild. Speaking generally the conjunctival secretion is markedly bactericidal, and as antiseptic lotions may destroy this power it is better to trust to aseptic rath[illegible]r than antiseptic precautions.

Pusey[6] points out that many of the organisms found in the human conjunctiva cannot live in the conjunctiva of the lower animals, and that whilst a healthy conjunctiva does not absorb toxins, an injured membrane does so immediately and becomes inflamed. He described chalazion as an infectious bacterial process excited by an organism of the xerosis type. The conjunctiva it has been proved may be the starting point of a general infection such as pneumococcic fever. Ulcer of the cornea he showed may be due to gonococci, pneumococci, or staphylococci.

**Bacteriology of the Blood.**—Houghton[7] has made an investigation of blood in three cases of puerperal sepsis. His article affords a fairly complete review of the literature relating to blood infection, and the various methods of technique which have been adopted, viz., the method of obtaining a drop by pricking the finger—Scheurlen's glass tube method, and Sittman's sterile syringe. His conclusions are as follows: (1) Bacteria and cocci may be present in the blood of healthy persons; (2) bacteriological examination of the blood is of little or no value for purposes of diagnosis, prognosis, or treatment; (3) a positive result is of little service, as a second examination may prove blank, or the infection may have been cryptogenic, or the organism found may not be the primary infective agent, or may be only one of two or more infective agents; (4) negative results cannot be relied on, as the quantity of blood taken is very small. Houghton's conclusions will not be generally accepted in this country, but he brings out several interesting points. The blood in the cases detailed was abstracted from a vein by means of a hypodermic needle fitted to a Eulenmeyer flask. About 1cc. of blood was mixed with 15cc. of culture medium. As a medium for culture he suggests the following: Water, 100gms.; meat extract, 5gms.; peptone, 30gms.; glucose, 5gms.; glycerine, 50gms. Two of the cases of septic metritis showed a general blood infection, one by staphylococcus and one by streptococcus. The third case gave negative results. A small incision over the vein may be necessary in order to obtain the blood, and such a procedure would tend to lessen the risk of accidental contamination. The author believes that pyogenetic organisms may enter the blood of healthy persons through the tonsil, gut, respiratory tract, or skin. He is also of opinion that the normal kidney may secrete organisms, bacilli and cocci, if such are present in the blood stream.

1 Deut. Med. Woch., Sept. 24, 1903. 2 Amer. Jour. Med. Sci., Dec. 12, 1902. 3 Brit. Med. Jour., Aug. 29, 1903. 4 John Hop. Hosp. Reps., Vol. X., No. 1-2. 5 Jour. Amer. Med. Ass., Oct. 3, 1903. 6 Ibid. 7 The Postgraduate, Sept., 1903.

